# Early Predictors of Objective Response in Patients with Hepatocellular Carcinoma Undergoing Lenvatinib Treatment

**DOI:** 10.3390/cancers12040779

**Published:** 2020-03-25

**Authors:** Issei Saeki, Takahiro Yamasaki, Satoyoshi Yamashita, Tadasuke Hanazono, Yohei Urata, Takakazu Furutani, Yuichiro Yokoyama, Toshiyuki Oishi, Masaki Maeda, Teruaki Kimura, Yurika Kotoh, Ryo Sasaki, Takashi Miyaji, Takashi Oono, Yuki Aibe, Takuro Hisanaga, Takuya Iwamoto, Toshihiko Matsumoto, Isao Hidaka, Tsuyoshi Ishikawa, Taro Takami, Isao Sakaida

**Affiliations:** 1 Department of Gastroenterology and Hepatology, Yamaguchi University Graduate School of Medicine, Ube, Yamaguchi 755-8505, Japan; issaeki@yamaguchi-u.ac.jp (I.S.); g030ub@yamaguchi-u.ac.jp (Y.K.); ryo0530@yamaguchi-u.ac.jp (R.S.); w032ub@yamaguchi-u.ac.jp (T.M.); toono@yamaguchi-u.ac.jp (T.O.); y.aibe@yamaguchi-u.ac.jp (Y.A.); t-hisa01@yamaguchi-u.ac.jp (T.H.); t_iwamot@yamaguchi-u.ac.jp (T.I.); tm0831@yamaguchi-u.ac.jp (T.M.); isao-h@yamaguchi-u.ac.jp (I.H.); tsu0920@yamaguchi-u.ac.jp (T.I.); t-takami@yamaguchi-u.ac.jp (T.T.); sakaida@yamaguchi-u.ac.jp (I.S.); 2 Department of Oncology and Laboratory Medicine, Yamaguchi University Graduate School of Medicine, Ube, Yamaguchi 755-8505, Japan; 3 Department of Gastroenterology, Shimonoseki Medical Center, Shimonoseki, Yamaguchi 750-0061, Japan; yamashita-satoyoshi@shimonoseki.jcho.go.jp; 4 Department of Gastroenterology, Saiseikai Shimonoseki General Hospital, Shimonoseki, Yamaguchi 759-6603, Japan; t_hana_zono@yahoo.co.jp; 5 Department of Gastroenterology, Yamaguchi Rosai Hospital, Sanyo-Onoda, Yamaguchi 756-0095, Japan; yoheiurata@yahoo.co.jp; 6 Department of Gastroenterology, Shuto General Hospital, Yanai, Yamaguchi 742-0032, Japan; takakazu@bk9.so-net.ne.jp; 7 Department of Gastroenterology, Tokuyama Central Hospital, Syunan, Yamaguchi 745-8522, Japan; 824yokoyama@gmail.com; 8 Department of Gastroenterology, Kokura Memorial Hospital, Kokura, Fukuoka 802-8555, Japan; toshi.ooishi@gmail.com; 9 Department of Gastroenterology, Nagato General Hospital, Nagato, Yamaguchi 759-4194, Japan; poohsuke_shiba@yahoo.co.jp; 10 Department of Gastroenterology, Yamaguchi Prefectural Grand Medical Center, Yamaguchi, Yamaguchi 747-8511, Japan; burnoutnash@ymghp.jp

**Keywords:** hepatocellular carcinoma, lenvatinib, molecular targeted agent, objective response

## Abstract

There are limited reports regarding early predictors of objective response (OR) in patients with hepatocellular carcinoma (HCC) treated with lenvatinib. This retrospective study including 70 patients aimed to investigate the efficacy of hepatic biochemical markers. Changes in tumor marker (alpha-fetoprotein (AFP)/des-gamma-carboxy prothrombin (DCP)) levels and albumin–bilirubin (ALBI) score between the baseline value and that estimated one month after treatment were evaluated. We identified several predictors of OR, including changes in tumor marker levels. The OR rate calculated using modified Response Evaluation Criteria in Solid Tumor (mRECIST) was 41.4%. Response was defined as a reduction in AFP and DCP levels of ≥40% from baseline. OR was significantly associated with AFP response, but not with DCP. Predictors of OR were evaluated in two groups (high-AFP group: baseline AFP ≥ 10 ng/mL; low-AFP group: remaining patients). A multivariate analysis identified AFP response (odds ratio, 51.389; *p* = 0.001) and ALBI score (odds ratio, 6.866; *p* = 0.039) as independent predictors of OR in the high-AFP and low-AFP groups, respectively. Changes in the ALBI score indicated deterioration in both responders and non-responders, with a significant difference in non-responders (*p* = 0.003). AFP response, baseline ALBI score, and change in the ALBI score were early predictors of OR in patients with HCC undergoing lenvatinib treatment.

## 1. Introduction

Systemic therapy for hepatocellular carcinoma (HCC) has changed drastically since the introduction of sorafenib, a molecular-targeted agent (MTA), in 2007. Recent success of clinical trials undertaken to study MTAs, including regorafenib, lenvatinib, cabozantinib, and ramucirumab, may promote a change in the treatment strategy in patients at the intermediate and advanced stages of HCC [1]. The phase III REFLECT study demonstrated non-inferiority of lenvatinib to sorafenib in patients with advanced HCC [2]. Therefore, in addition to sorafenib, lenvatinib is also recommended as the standard first-line systemic therapy for HCC, according to several guidelines [3,4,5,6]. Following its approval for use in clinical practice in Japan in March 2018, lenvatinib has been administered in real-world practice, not only as a first-line treatment, but also as part of second- or third-line therapy for HCC. In contrast, the phase III RESORCE study demonstrated that sequential administration of sorafenib, followed by that of regorafenib, extended patient survival (median survival time (MST): 26.0 months for sorafenib–regorafenib vs. 19.6 months for sorafenib–placebo) [7]. Furthermore, according to a post-hoc analysis of the REFLECT study, patients treated with lenvatinib, who received subsequent anticancer medication (most common being sorafenib), had longer median overall survival (OS) than those who did not receive subsequent medication (20.8 months for sequential therapy vs. 11.5 months for non-sequential therapy) [8]. The use of two or more MTAs sequentially may yield good OS, making it important to administer lenvatinib accordingly and to identify early predictors of objective response (OR) in patients with HCC undergoing lenvatinib treatment. Nevertheless, there have been a few reports regarding early predictors of OR in such patients [9,10].

Treatment response is generally evaluated using imaging modalities. In addition, tumor markers have been used as biomarkers of treatment response in patients with HCC receiving various treatments [11,12,13,14]. However, only one study has shown a relationship between early tumor marker response and radiographic OR in patients with HCC undergoing lenvatinib treatment [10]. Therefore, this study aimed to identify early predictors, including tumor markers, of OR in patients with HCC undergoing lenvatinib treatment.

## 2. Results

### 2.1. Patient Characteristics

Patient profiles are summarized in Table 1. Seventy patients (50 men and 20 women) were included in the study. The mean age and weight of the cohort were 72.8 years and 57.8 kg, respectively. Causes of HCC were hepatitis C, hepatitis B, alcohol consumption, and others in 31, 14, 7, and 18 patients, respectively. In total, 57 and 13 subjects were classified as Child–Pugh class A and B patients, respectively. Furthermore, 18, 48, and 4 patients were found to have an albumin–bilirubin (ALBI) grade [15] of 1, 2, and 3, respectively. According to the Barcelona Clinic Liver Cancer (BCLC) staging system [16], 3, 29, and 38 patients were classified as having BCLC stages A, B, and C, respectively. Lenvatinib was introduced as the treatment drug for three patients with BCLC stage A, who could not receive local therapy (local ablation and transcatheter arterial chemoembolization TACE) due to technical constraints and for 29 patients with BCLC stage B, who met TACE refractoriness criteria (*n* = 24) or were considered unsuitable for this therapeutic modality (*n* = 5) [17]. In total, 15 (21.4%) and 19 (27.1%) patients had developed macrovascular invasion (MVI) and extrahepatic spread (EHS), respectively. Median number and size of tumors were 4.0 and 30.0 mm, respectively. Median alpha-fetoprotein (AFP) and des-gamma-carboxy prothrombin (DCP) levels were 14.5 ng/mL (3.9–603.6 ng/mL) and 514.5 mAU/mL (50.3–5798.5 mAU/mL), respectively. Six patients (8.6%) had a history of receiving treatment with MTAs. The median observation period was 8.9 (5.3–11.8) months.

### 2.2. Relationship between Treatment Response and Response of Tumor Markers

The median duration for evaluating the best treatment response using imaging modalities was 2.7 (1.7–3.3) months. The objective response rate (ORR) and disease control rate (DCR) were 41.4% (29/70) and 68.6% (48/70), respectively (Table 2). We examined the relationship between imaging treatment response and rate of change in AFP and DCP levels in patients with HCC who had baseline levels greater than or equal to the upper limit of their respective normal range. The upper limit of the normal range for AFP is 10 ng/mL and for DCP is 40 mAU/mL. Moreover, 41 and 51 patients had baseline AFP and DCP values ≥ 10 ng/mL (high-AFP group) and ≥ 40 mAU/mL (high-DCP group), respectively. The cut-off value of the rate of change in AFP level was determined using a receiver-operating characteristic (ROC) analysis with a Youden index of 0.396 and sensitivity and specificity levels of 100.0% and 77.8%, respectively (Supplementary Figure S1). Therefore, we defined AFP response as a reduction of ≥40% from its baseline level. Conversely, as it was impossible to determine an optimal cut-off value for the rate of change in DCP level, a DCP response was defined as a reduction in its baseline level by ≥40% (similar to that used for defining AFP response). In the high-AFP group, ORRs were 68.4% (*n* = 19) and 7.1% (*n* = 22) among AFP responders and non-responders, respectively, which were significantly different (*p* < 0.001) (Table 2). Additionally, a significant difference in DCR was observed between AFP responders and non-responders (84.2% vs. 36.0%, *p* = 0.009). In the high-DCP group, no significant differences were noted between DCP responders (*n* = 9) and non-responders (*n* = 42) in relation to ORR and DCR (Table 2, one patient was excluded from the DCP analysis because of warfarin use). 

**Figure 1:**
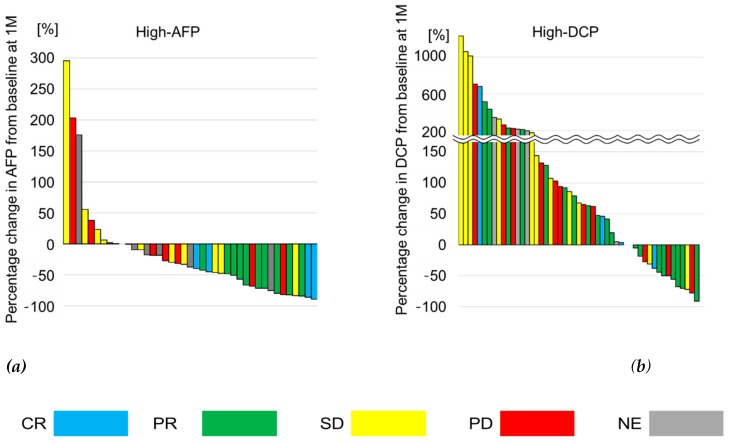
Changes in alpha-fetoprotein (AFP) and des-gamma-carboxy prothrombin (DCP) levels from baseline in the high-AFP group and the high-DCP group. (**a**) The median rate of change in AFP was −69.0% (−47.1% to −82.6%) in imaging responders (blue and green), while it was −17.7% (−37.2% to +0.5%) in imaging non-responders (yellow, red, and gray). (**b**) The median rate of change in DCP was +30.6% (−42.8% to +118.9%) in imaging responders and +107.6% (−4.7% to +262.3%) in imaging non-responders.

Waterfall plots of changes in tumor marker levels in both high-AFP and high-DCP groups are shown in Figure 1. Median rate of change in AFP level was −69.0% (−47.1% to −82.6%) in responders (complete response (CR) + partial response (PR)) detected on imaging, while it was −17.7% (−37.2% to +6.5%) in imaging non-responders (stable disease (SD) + progressive disease (PD) + no evaluation (NE)). In contrast, the median rate of change in DCP level was +30.6% (−42.8% to +118.9%) and +107.6% (−4.7% to +262.3%) in imaging responders and non-responders, respectively. Thus, the rate of change in DCP level showed no significant correlation to treatment response, as determined using imaging.

### 2.3. Predictors of Objective Response

**Figure 2:**
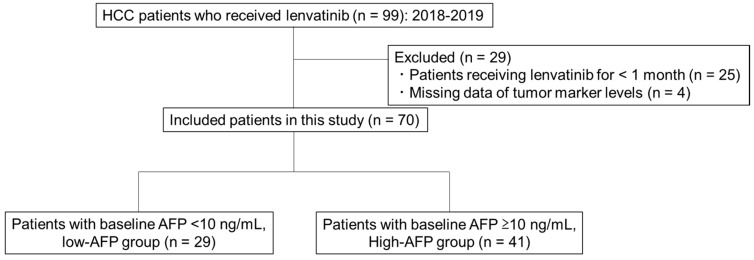
Study chart. In total, 99 patients were treated with lenvatinib at various centers. Of them, 70 patients met the inclusion criteria. They were divided into the high-alpha-fetoprotein (AFP) group (baseline AFP ≥ 10 ng/mL; *n* = 41) and the low-AFP group (baseline AFP < 10 ng/mL; *n* = 29).

We divided patients into the high-AFP (baseline AFP ≥ 10 ng/mL) and low-AFP (baseline AFP < 10 ng/mL) groups (Figure 2) and evaluated predictors of OR in each group. As shown in Supplementary Table S1, with the exception of AFP and DCP levels, no significant differences in patient characteristics were observed between the two groups.

Table 3 shows results of univariate and multivariate analyses of the high-AFP group (*n* = 41). A univariate analysis for each factor affecting OR, including AFP response and ALBI score, was performed. The cut-off level for ALBI score was set at −2.44 according to the ROC analysis (area under the curve (AUC), 0.643; sensitivity, 58.6%; specificity, 73.2%), while those for tumor size and number were set at median values. Finally, we used 200 ng/mL and 40 mAU/mL as cut-off values for AFP and DCP levels at baseline, respectively, as suggested by the Asia Pacific Association for the Study of the Liver HCC guideline [18]. 

Factors analyzed in the univariate analysis were sex (male/female), age (<65 years/≥65 years), body weight (<60 kg/≥60 kg), performance status (0/1–), ALBI score (<−2.44/≥−2.44), BCLC staging (A,B/C), tumor size (<30 mm/≥30 mm), tumor number (<4/≥4), EHS (absence/presence), MVI (absence/presence), AFP (<200 ng/mL/≥200 ng/mL), DCP (<40 mAU/mL/≥40 mAU/mL), AFP response (yes/no), and DCP response (yes/no). Of these, AFP response was a significant factor (*p* < 0.001). A multivariate analysis performed using factors found to be significant (*p*-value ≤ 0.1; sex, ALBI score, and AFP response) in the univariate analysis revealed that AFP response was the only significant predictor of OR (odds ratio, 51.389; 95% confidence interval (CI), 4.888–540.281; *p* = 0.001). In the low-AFP group (*n* = 29), the univariate analysis was performed for the following factors affecting OR: sex (male/female), age (<65 years/≥65 years), body weight (<60 kg/≥60 kg), performance status (0/1–), ALBI score (<−2.44/≥−2.44), BCLC staging (A,B/C), tumor size (<30 mm/≥30 mm), tumor number (<4/≥4), EHS (absence/presence), MVI (absence/presence), and DCP (<40 mAU/mL/≥40 mAU/mL) (Table 4). Consequently, ALBI score was found to be a significant factor (*p* = 0.042). A subsequent multivariate analysis of significant factors (*p*-value ≤ 0.1; ALBI score and DCP) demonstrated ALBI score (odds ratio, 6.866; 95% CI, 1.098–42.923; *p* = 0.039) as an independent predictor of OR.

### 2.4. Change in ALBI Score

**Figure 3:**
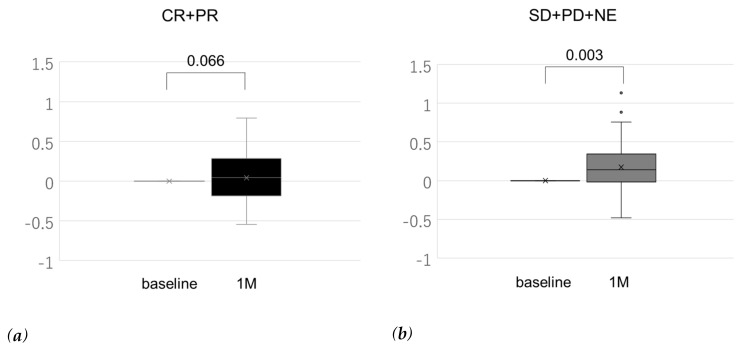
Change in albumin–bilirubin (ALBI) score from baseline to one month after treatment initiation according to the imaging response. (**a**) In imaging responders, the ALBI score did not show significant worsening (+0.11 (−0.14 to +0.35), *p* = 0.066). (**b**) In imaging non-responders, the ALBI score showed significant deterioration (+0.14 (−0.02 to +0.34), *p* = 0.003). The x-mark and black dot indicate the mean and outlier beyond 1.5× interquartile range, respectively. CR, complete response; PR, partial response; SD, stable disease; PD, progressive disease; NE, no evaluation.

Figure 3 shows the relationship between treatment response and change in ALBI score from baseline to that measured one month after treatment initiation. The ALBI score worsened significantly in all patients (+0.14 (−0.07 to +0.35), *p* < 0.001)). Interestingly, the ALBI score showed significant deterioration in imaging non-responders (+0.14 (−0.03 to +0.34), *p* = 0.003). While the ALBI score also worsened in therapy responders, the difference was not significant (+0.11 (−0.14 to +0.35), *p* = 0.066).

## 3. Discussion

In this study, we showed the effect of AFP response and ALBI score on OR in patients with HCC undergoing lenvatinib treatment. Changes in the DCP level did not correlate with OR, as previously reported [10]. As shown in Figure 1b, the DCP level at one month after treatment increased in 35/51 (68.6%) patients in the high-DCP group. As lenvatinib exerts a strong anti-angiogenic effect, DCP induction may occur not only due to tumor progression, but also following hypoxia stimulation [19]. This phenomenon has also been reported in patients with HCC undergoing sorafenib treatment [20,21]. Although tumor markers have been used as biomarkers of treatment response in patients with HCC, those with normal baseline tumor marker levels were excluded [12,13,14]. Therefore, by dividing patients into two groups using the upper limit value of the normal range of the AFP level as the cut-off, we evaluated the predictors of OR in each group.

In the high-AFP group, we demonstrated AFP response (odds ratio, 51.389; *p* = 0.001) as an independent predictor of OR in the multivariate analysis. In our study, median rate of change in the AFP level was −69.0% in imaging responders versus −17.7% in imaging non-responders. A previous study also showed that the median AFP change at one month was −63% in responders and −22% in non-responders, which was a significant difference, although an AFP response was not defined [10]. Therefore, we considered that an assessment of changes in the AFP level in response to treatment was necessary for clinical practice. We defined AFP response as a reduction of ≥40% from the baseline according to ROC analysis with Youden index. Our definition of AFP response may be appropriate as an early predictor of OR.

In the low-AFP group, our multivariate analysis identified ALBI score (odds ratio, 6.866; *p* = 0.039) as an independent predictor of OR. This means that an ALBI score less than −2.44, indicative of modified ALBI (mALBI) grades 1 and 2a (mALBI 1: −2.82 to −2.52, mALBI 2a: −2.46 to −2.31) [22], was predictive of a higher OR. Ueshima et al. also reported that ALBI grade 1 was an independent predictor of OR [23]. Another study showed that maintaining relative dose intensity during the initial eight weeks of lenvatinib treatment was significantly associated with a higher OR [9]. In this study, we evaluated the rate of discontinuation of lenvatinib between patients with ALBI score less than −2.44 and those with ALBI score greater than or equal to –2.44 and found that patients in the former group had continued lenvatinib treatment for a significantly longer period than those in the latter (13.3 vs. 6.1 months, *p* = 0.014; Supplementary Figure S2). Therefore, patients with good liver function may have demonstrated a higher OR as a consequence of receiving lenvatinib treatment over longer duration. In addition, we studied the relationship between treatment response as indicated by imaging studies and change in the ALBI score from baseline to that measured one month after lenvatinib initiation. The ALBI score is a quantitative performance indicator (as compared to the Child–Pugh classification), making it a useful tool for the comparison of patients’ status before and after receiving treatment. In all patients, the ALBI score had significantly worsened (+0.14, *p* < 0.001). We compared the change in ALBI score of imaging responders with that of non-responders as indicated by imaging. While the ALBI score had deteriorated in both groups, the change was significant in therapeutic non-responders (+0.14, *p* = 0.003), while insignificant in responders (+0.11, *p* = 0.066). Although the worsening of mALBI grades 1, 2a, and ≥2b from baseline to that estimated one month after receiving lenvatinib treatment was reported to deteriorate significantly regardless of the baseline grade, no previous reports have indicated a relationship between treatment response and change in the ALBI score [24]. In this study, the baseline ALBI score differed significantly between imaging responders and non-responders (−2.42 ± 0.49 vs. −2.17 ± 0.54, *p* = 0.047; Supplementary Table S2). In other words, responders had better baseline ALBI scores than non-responders as indicated by imaging. Thus, we matched liver function (ALBI grade) using propensity scores (Supplementary Table S3) to comparatively assess change in the ALBI score. As expected, the ALBI score worsened significantly in imaging non-responders after one month of receiving treatment (+0.18 (0 to 0.06), *p* = 0.006), but the deterioration was insignificant in imaging responders (+0.10 (−0.16 to 0.37), *p* = 0.103) (Supplementary Figure S3). Therefore, change in the ALBI score may be also an early predictor of OR.

A sub-analysis of the RESORCE study demonstrated the efficacy of MTA sequential therapy in patients with HCC [7]. In addition, Hiraoka et al. reported that an important clinical predictor in MTA sequential therapy is good liver function (mALBI grade 1/2a) [25]. Interestingly, this result was similar to our finding of ALBI score less than −2.44 being a predictor of OR in the low-AFP group. Therefore, we consider that lenvatinib treatment should be started in patients with good baseline liver function.

Based on these findings, AFP response in patients with baseline AFP ≥ 10 ng/mL, baseline ALBI score in patients with level <10 ng/mL, and change in ALBI score from baseline to that estimated one month post-treatment may have a great impact on OR in patients with HCC undergoing lenvatinib treatment. As the median time interval for evaluating the best treatment response was 2.7 months, we believe that these predictors provide meaningful information to estimate OR that precedes the timing at which prognostic imaging needs to be performed. It may be possible to switch the treatment of non-responders from lenvatinib to another possibly effective MTA, earlier. However, the retrospective nature of the study makes it difficult to draw such a conclusion. This also necessitates evaluating the relationship between tumor marker response and radiographic response at one month post-treatment.

This study has several limitations. First, although study subjects were recruited from various centers, the cohort size was small. Another shortcoming is the retrospective study design. However, our assessment of patients with HCC undergoing lenvatinib treatment is a novel concept. Future prospective studies of a large population are required to validate our findings. Second, the short observation period was inadequate to evaluate its effect on OS. At this time, the OS of imaging responders tended to be longer than that of imaging non-responders (MST: not reached for responders, 12.7 months for non-responders, *p* = 0.077, data not shown). Although OR was assessed using the modified Response Evaluation Criteria in Solid Tumors (mRECIST), predicting survival in patients receiving loco-regional treatments [4] and OR to systemic therapies is a controversial topic because of its effect on survival. A sub-analysis of the REFLECT study demonstrated that OR was an independent prognostic factor [26] and that administration of sequential therapy after lenvatinib improved OS as compared to that observed with administration of non-sequential therapy [8]. However, further evaluation in the clinical practice setting is indicated.

## 4. Materials and Methods 

### 4.1. Patients

This was a retrospective cohort study of 99 consecutive patients with HCC, who were treated with lenvatinib at nine institutions, including the Yamaguchi University Hospital, between May 2018 and March 2019. The inclusion criteria were as follows: (1) unresectable HCC confirmed by pathological or imaging diagnosis, (2) lenvatinib treatment for more than one month, and (3) measurement of the levels of the tumor markers AFP and DCP before and at one month after treatment initiation. Consequently, 70 patients were included in this study (Figure 2). The observation period was set until 31 July 2019. This study protocol (H26-109) was approved by the Institutional Review Board of the Yamaguchi University Hospital and of each participating institution and was conducted in compliance with the ethical principles in the Declaration of Helsinki. No informed consent was needed because of the retrospective study design.

### 4.2. Treatment Protocol

Lenvatinib (Lenvima®; Eisai Co., Ltd, Tokyo, Japan) was administered orally to patients with unresectable HCC. The initial dose of lenvatinib was determined according to a patient’s body weight and liver function. Patients who weighed ≥60 kg and were classified as Child–Pugh A were started at a dose of 12 mg once daily, those who weighed <60 kg and belonged to Child–Pugh A class were started at a dose of 8 mg once daily, and those found to meet Child–Pugh B criteria were started at a dose of 8 mg. When adverse events (AEs) occurred during treatment, the dosage was reduced or treatment was interrupted. Patients received lenvatinib until either radiological tumor progression or onset of intolerable AEs. 

### 4.3. Evaluation of Treatment Response, Tumor Markers, and Liver Function

Treatment response was evaluated using dynamic computed tomography or magnetic resonance imaging according to the mRECIST criteria [27]. Tumor assessment was performed every two to three months. The best response was used for response evaluation, OR (CR + PR), and disease control (CR + PR + SD) at each institution. A subset of patients who died before tumor assessment was defined as NE. Serum AFP and DCP levels were measured at baseline (before lenvatinib administration) and at one month after the start of treatment. Liver function was evaluated using the Child–Pugh classification and the ALBI scoring system [15]. ALBI score was calculated using serum albumin and bilirubin values at baseline and at one month after the administration of lenvatinib as follows: ALBI score = (log 10 bilirubin (µmol/L) × 0.66) + (albumin (g/L) × −0.085).

Rate of change in tumor marker (AFP/DCP) levels at one month was defined as follows: percentage change in tumor marker levels at one month = tumor marker level at one month/tumor marker level at baseline. Moreover, change in the ALBI score at one month was defined as follows: change in ALBI score = ALBI score at one month − ALBI score at baseline.

### 4.4. Statistical Analysis

Data were expressed as mean and standard deviation or median and interquartile range. A statistical analysis between any two groups was performed using a paired or an unpaired t-test. Categorical variables were analyzed using chi-square test or Fisher’s exact test. The best cut-off value of change in tumor marker levels or for the ALBI score in the ROC curve was determined using the Youden index. Univariate and multivariate analyses of OR were performed using logistic regression analyses, and the results were presented as odds ratio with 95% CIs. Statistical significance was defined as *p* < 0.05. All analyses were performed using the JMP software package v13.0 (SAS Institute, Cary, NC, USA).

## 5. Conclusions

The change in AFP response from baseline to that at one month, baseline ALBI score, and change in ALBI score from baseline to that at one month were early predictors of OR in patients with HCC undergoing lenvatinib treatment. These predictors, consisting of three commonly used factors (AFP, bilirubin, and albumin), may help with therapeutic assessment of patients with HCC receiving lenvatinib.

## Figures and Tables

**Table 1:** Patient characteristics.

Factor	Total
Age	72.8 ± 9.1
Sex male/female	50/20
BW (kg)	57.8 ± 12.1
Etiology C/B/Alc/N	31/14/7/18
Child–Pugh class A/B	57/13
ALBI score	−2.27 ± 0.53
ALBI grade 1/2/3	18/48/4
PS 0/1/2	58/11/1
BCLC ^a^ A/B/C	3/29/38
MVI absence/presence	55/15
EHS absence/presence	51/19
Tumor number	4 (1–8)
Tumor size (mm)	29.0 (20.0–45.3)
AFP (ng/mL)	14.5 (3.9–603.6)
DCP (mAU/mL)	271.0 (35.6–2158.5)
Prior treatment (+/−)	61/9
Prior treatment of MTA (+/−): SORA/REGO	6/64:6/3

Values are expressed as numbers, mean ± standard deviation, or median (interquartile range). BW, body weight; C, hepatitis C virus; B, hepatitis B virus; Alc, alcohol; N, non-B, non C; ALBI, albumin–bilirubin; PS, performance status; MVI, macrovascular invasion; EHS, extrahepatic spread; AFP, alpha-fetoprotein; DCP, des-gamma-carboxy prothrombin; MTA, molecular-targeted agent; SORA, sorafenib; REGO, regorafenib. ^a^ BCLC, Barcelona Clinic Liver Cancer staging.

**Table 2:** Relationship between treatment response and response of tumor markers.

Response ^a^	CR	PR	SD	PD	NE	ORR	DCR
Total (n = 70)	6	23	19	15	7	41.4 %	68.6 %
**High-AFP group (AFP ≥ 10ng/mL)**							
Total (n = 41)	3	11	11	10	6	34.2%	61.0%
AFP responder b (n = 19)	2	11	3	2	1	68.4%]_*_	84.2%]_+_
AFP non-responder (n = 22)	1	0	8	8	5	7.1%	36.0%
**High-DCP group (DCP ≥ 40ng/mL)**							
Total (n = 51)	4	20	11	12	4	47.1%	68.6%
DCP responder b (n = 9)	0	6	1	2	0	66.7%	78.8%
DCP non-responder (n = 42)	4	14	10	10	4	42.9%	66.7%

CR, complete response; PR, partial response, SD, stable disease; PD, progressive disease; NE, no evaluation; ORR, objective response rate; DCR, disease control rate; AFP, alpha-fetoprotein; DCP, des-gamma-carboxy prothrombin. ^a^ Evaluated by modified RECIST; ORR = (CR + PR)/(CR + PR + SD + PD + NE); DCR = (CR + PR + SD)/(CR + PR + SD + PD + NE); ^b^ AFP and DCP responses were assessed one month after lenvatinib induction; a positive-response was defined as a reduction of ≥40% from baseline; *, *p* < 0.001; +, *p* = 0.009.

**Table 3:** Univariate and multivariate analyses for factors affecting objective response in the high-alpha-fetoprotein group.

Factors	Univaritate Analysis			Multivariate Analysis		
	Odds Ratio	95% CI	*p*-Value	Odds ratio	95% CI	*p*-Value
Sex (M/F)	4.125	0.767–22.184	0.099	4.825	0.560–41.587	0.152
Age (<65 years/≥65 years)	2.857	0.736–11.086	0.129			
BW (<60 kg/≥60 kg)	3.000	0.549–16.379	0.245			
PS (0/1–)	2.100	0.374–11.807	0.400			
ALBI (<−2.44/≥−2.44)	3.167	0.827–12.126	0.093	2.714	0.371–19.854	0.325
BCLC ^a^ A,B/C	1.700	0.460–6.280	0.426			
Tumor size (mm) (<30/≥30)	1.938	0.513–7.319	0.329			
Tumor number (<4/≥4)	1.091	0.295–4.033	0.896			
EHS absence/presence	2.521	0.568–11.181	0.224			
MVI absence/presence	1.053	0.254–4.371	0.944			
AFP (ng/mL) (<200/≥200)	0.808	0.220–2.964	0.748			
DCP (mAU/mL) (<40/≥40)	0.556	0.096–3.207	0.511			
AFP response ^b^	45.500	4.907–421.933	<0.001	51.389	4.888–540.281	0.001
DCP response ^b^	0.917	0.146–5.757	0.926			

M, male; F, female; BW, body weight; PS, performance status; ALBI, albumin–bilirubin; MVI, macrovascular invasion; EHS, extrahepatic spread; AFP, alpha-fetoprotein; DCP, des-gamma-carboxy prothrombin; CI, confidence interval; ^a^ BCLC, Barcelona Clinic Liver Cancer staging. ^b^ AFP and DCP responses were assessed one month after lenvatinib induction; a positive response was defined as a reduction of ≥40% from baseline.

**Table 4:** Univariate and multivariate analyses for factors affecting objective response in the low-alpha-fetoprotein group.

Factors	Univaritate Analysis			Multivariate Analysis			
	Odds Ratio	95% CI	*p*-Value		Odds Ratio	95% CI	*p*-Value
Sex (M/F)	0.750	0.135–4.165	0.742				
Age (<65 years/≥65 years)	0.655	0.134–3.187	0.600				
BW (<60 kg/≥60 kg)	0.857	0.198–3.713	0.837				
PS (0/1–)	0.500	0.040–6.128	0.590				
ALBI (<−2.44/≥−2.44)	5.500	1.065–28.416	0.042		6.866	1.098–42.923	0.039
BCLC ^a^ A,B/C	0.656	−0.151–2.843	0.573				
Tumor size (mm) (<30/≥30)	0.667	0.153–2.903	0.589				
Tumor number (<4/≥4)	3.667	0.771–17.429	0.102				
EHS absence/presence	0.667	0.094–4.373	0.685				
MVI absence/presence	0.500	0.040–6.218	0.590				
DCP (mAU/mL) (<40/≥40)	0.256	0.048–1.282	0.098		0.191	0.029–1.266	0.086
AFP response ^b^	1.333	0.240–7.405	0.742				
DCP response ^b^	-	-	-				

M, male; F, female; BW, body weight; PS, performance status; ALBI, albumin-bilirubin; MVI, macrovascular invasion; EHS, extrahepatic spread; AFP, alpha-fetoprotein; DCP, des-gamma-carboxy prothrombin; CI, confidence interval; ^a^ BCLC, Barcelona Clinic Liver Cancer staging; ^b^ AFP and DCP responses were assessed after one months after lenvatinib induction; a positive response was defined as a reduction of ≥ 40% from baseline.

## Supplementary Materials

The following are available online at https://www.mdpi.com/2072-6694/12/4/779/s1, Table S1: Patient characteristics between low-alpha-fetoprotein (AFP) group and high-AFP group. Table S2: Patient characteristics between imaging responders and non-responders. Table S3: Patient characteristics after propensity score matching between imaging responders and non-responders. Figure S1: The receiver-operating characteristic (ROC) analysis for objective response of the rate of change in alpha-fetoprotein. Figure S2: Cumulative time to treatment failure classified as two groups according to baseline albumin–bilirubin (ALBI) score, those with <−2.44 and ≥2.44. Figure S3: Change in albumin–bilirubin (ALBI) score from baseline to one month after treatment initiation according to the imaging response after propensity score matching.
