# Supplementary material for: Early Predictors of Objective Response in Patients with Hepatocellular Carcinoma Undergoing Lenvatinib Treatment
Source: Cancers (Basel). 2020 Mar 25;12(4):779. doi: 10.3390/cancers12040779 (PMC7225985; doi:10.3390/cancers12040779)
Supplement: Supplementary file 1 [file cancers-12-00779-s001.pdf]

Supplementary Materilas

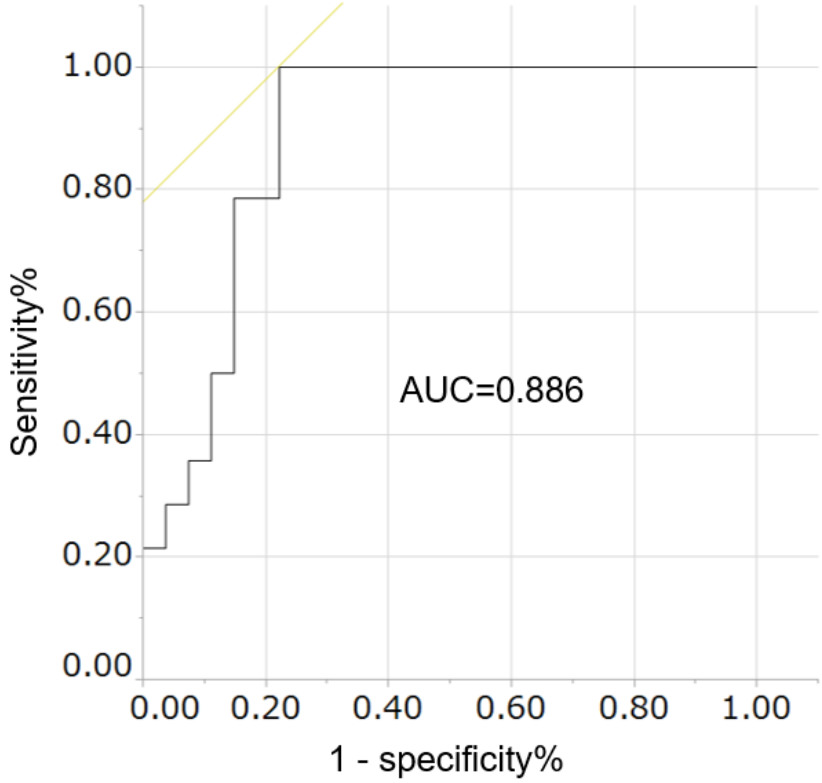

Figure S1. The receiver-operation characteristic (ROC) analysis for objective response of the rate of change in alpha-fetoprotein.

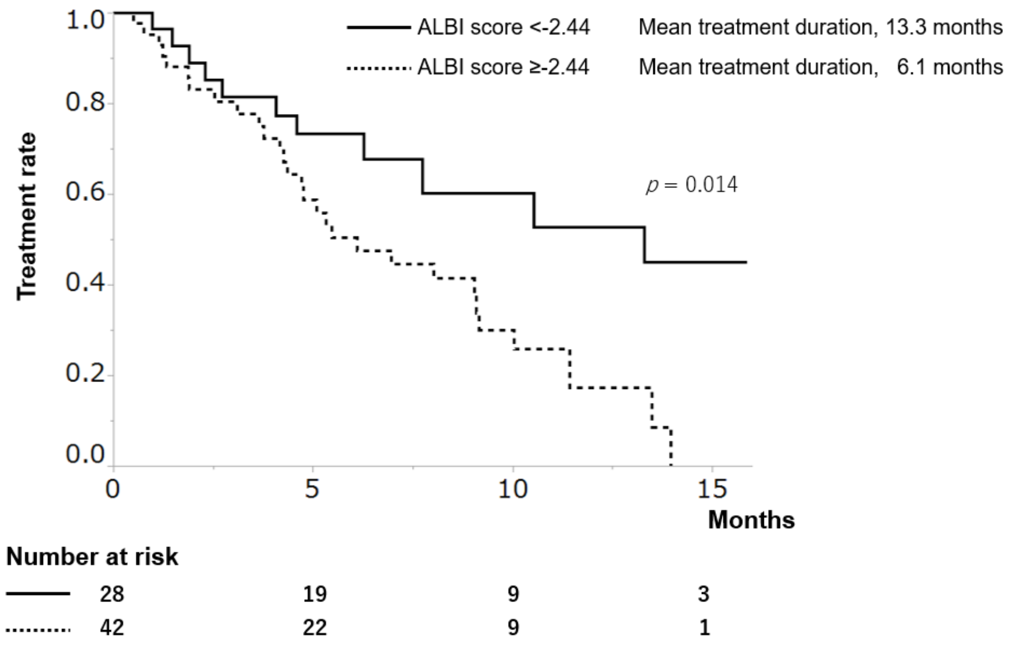

Figure S2 Cumulative time to treatment failure classified as two groups according to baseline albumin-bilirubin (ALBI) score, those with <−2.44 and ≥2.44.

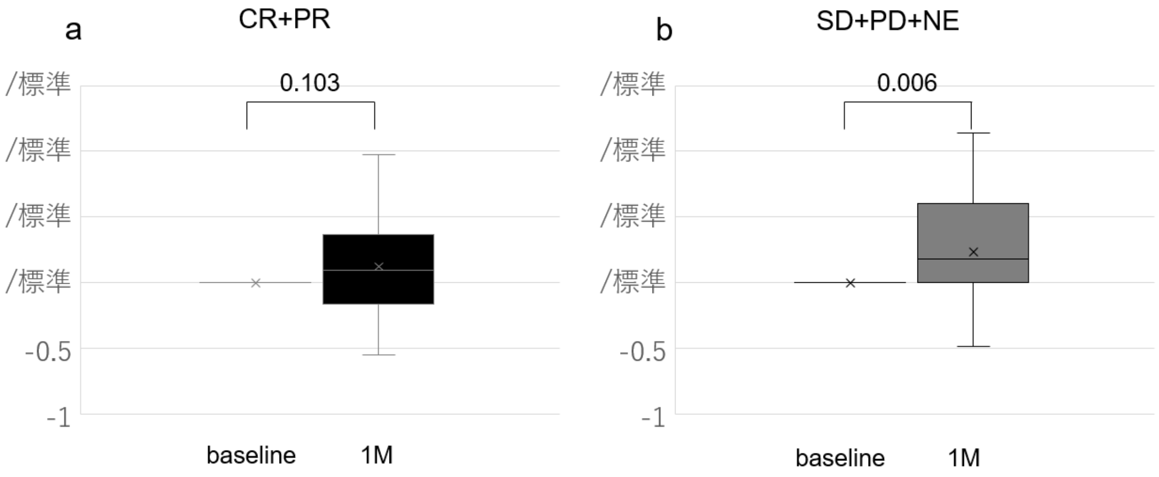

Figure S3. Change in albumin-bilirubin (ALBI) score from baseline to one month after treatment initiation according to the imaging response after propensity score matching.

Table S1. Patient characteristics between low-alpha-fetoprotein (AFP) group and high-AFP group.

Factor	Low-AFP Group	High-AFP Group	*p*-value
Age	73.8 ± 8.0	72.1 ± 9.8	0.452
Sex male/female	22/7	28/13	0.595
BW [kg]	57.9 ± 12.0	57.9 ± 12.4	0.988
Etiology C/B/Alc/N	12/5/3/9	19/9/4/9	0.841
Child-Pugh class A/B	23/6	34/7	0.761
ALBI grade 1/2/3	8/20/1	10/28/3	0.916
PS 0/1/2	26/2/1	32/9/0	0.126
BCLC [a] A/B/C	1/14/14	2/15/24	0.616
Vp 0/1/2/3/4	26/1/1/0/1	29/4/2/6/0	0.114
MVI absence/presence	26/3	29/12	0.078
EHS absence/presence	24/5	27/14	0.173
Tumor number	3 (1–7.5)	5 (2–9)	0.356
Tumor size [mm]	30.0 (20.5–43.5)	25.0 (17.0–54.0)	0.547
AFP [ng/mL]	3.3 (2.3–4.4)	360.0 (16.5–2074.0)	<0.001
DCP [mAU/mL]	108.0 (23.7–511.0)	514.5 (50.3–5798.5)	0.012
Prior treatment (+/−)	27/2	34/7	0.289
Prior treatment of MTA (+/−): SORA/REGO	1/28: 1/1	5/36: 5/2	0.389

Values are expressed as numbers, mean ± standard deviation, or median (interquatile range). C, Hepatitis C virus; B, Hepatitis B virus; Alc, Alcohol; N, non-B nonC; ALBI, albumin-bilirubin; MVI, Macrovascular invasion; EHS, Extrahepatic spread; AFP, alpha-fetoptrotein; DCP, des-gamma-carboxy prothrombin; SORA, sorafenib; REGO, regorafenib. [a] BCLC, The Barcelona Clinic Liver Cancer staging.

Table S2. Patient characteristics between imaging responders and non-responders.

Factor	Responder (29)	Non-Responder (41)	*p*-value
Age	72.6 ± 9.5	73.0 ± 8.8	0.882
Sex male/female	23/6	27/14	0.287
BW [kg]	58.3 ± 11.7	57.5 ± 12.6	0.782
Etiology C/B/Alc/N	11/6/2/10	20/8/5/8	0.490
Child-Pugh class A/B	26/3	31/10	0.213
ALBI score	−2.42 ± 0.49	−2.17 ± 0.54	0.047
ALBI grade 1/2/3	10/18/1	8/30/3	0.327
PS 0/1/2	25/3/1	33/8/0	0.249
BCLC [a] A/B/C	2/12/15	1/17/23	0.659
Vp 0/1/2/3/4	23/1/2/3/0	32/4/1/3/1	0.547
MVI absence/presence	23/6	32/9	1.000
EHS absence/presence	23/6	28/13	0.415
Tumor number	3 (1–6)	6 (2–10)	0.114
Tumor size [mm]	28 (18.5–44.0)	30.0 (20.0–45.5)	0.793
AFP [ng/mL]	9.4 (3.0–433.5)	16.7 (4.4–865.5)	0.198
DCP [mAU/mL]	254.0 (45.5–1076.5))	319.1 (28.8–3029.3)	0.908

Values are expressed as numbers, mean ± standard deviation, or median (interquatile range). C, Hepatitis C virus; B, Hepatitis B virus; Alc, Alcohol; N, non-B nonC; ALBI, albumin-bilirubin; MVI, Macrovascular invasion; EHS, Extrahepatic spread; AFP, alpha-fetoptrotein; DCP, des-gamma-carboxy prothrombin; [a] BCLC, The Barcelona Clinic Liver Cancer staging.

Table S3. Patient characteristics after propensity score matching between imaging responders and non-responders.

Factor	Responder (27)	Non-Responder (27)	*p* value
Age	72.8 ± 9,9	72.8 ± 9.1	0.989
Sex male/female	21/6	17/10	0.372
BW [kg]	58.3 ± 12.1	56.7 ± 11.1	0.609
Etiology C/B/Alc/N	11/6/1/9	16/3/3/5	0.245
Child-Pugh class A/B	24/3	20/7	0.294
ALBI score	−2.36 ± 0.45	−2.30 ± 0.56	0.635
ALBI grade 1/2/3	8/18/1	8/18/1	1.000
PS 0/1/2	23/3/1	23/4/0	0.465
BCLC [a] A/B/C	2/12/13	1/14/12	0.766
Vp 0/1/2/3/4	21/1/2/3/0	22/3/1/0/1	0.138
MVI absence/presence	21/6	22/5	1.000
EHS absence/presence	23/4	21/6	0.728

Tumor number	3 (1–6)	5 (1–10)	0.305
Tumor size [mm]	30.0 (20.0–46.0)	27.0 (20.0–40.0)	0.697
AFP [ng/mL]	9.4 (2.8–360.0)	15.4 (4.3–582.8)	0.359
DCP [mAU/mL]	254.0 (50.9–669.0))	197.5 (25.0–1481.3)	0.522

Values are expressed as numbers, mean ± standard deviation, or median (interquatile range). C, Hepatitis C virus; B, Hepatitis B virus; Alc, Alcohol; N, non-B nonC; ALBI, albumin-bilirubin; MVI, Macrovascular invasion; EHS, Extrahepatic spread; AFP, alpha-fetoptrotein; DCP, des-gamma-carboxy prothrombin; [a] BCLC, The Barcelona Clinic Liver Cancer staging.
